# Detection and explanation of anomalies in healthcare data

**DOI:** 10.1007/s13755-023-00221-2

**Published:** 2023-04-06

**Authors:** Durgesh Samariya, Jiangang Ma, Sunil Aryal, Xiaohui Zhao

**Affiliations:** 1 Institute of Innovation, Science and Sustainability, Federation University, Berwick, VIC Australia; 2 School of Information Technology, Deakin University, Geelong, VIC Australia; 3 Institute of Innovation, Science and Sustainability, Federation University, Ballarat, VIC Australia

**Keywords:** Outlying aspect mining, Healthcare, Outlier detection, Outlier explanation, Anomaly detection, Anomaly explanation

## Abstract

The growth of databases in the healthcare domain opens multiple doors for machine learning and artificial intelligence technology. Many medical devices are available in the medical field; however, medical errors remain a severe challenge. Different algorithms are developed to identify and solve medical errors, such as detecting anomalous readings, anomalous health conditions of a patient, etc. However, they fail to answer why those entries are considered an anomaly. This research gap leads to an outlying aspect mining problem. The problem of outlying aspect mining aims to discover the set of features (a.k.a subspace) in which the given data point is dramatically different than others. In this paper, we present a framework that detects anomalies in healthcare data and then provides an explanation of anomalies. This paper aims to effectively and efficiently detect anomalies and explain why they are considered anomalies by detecting outlying aspects. First, we re-introduced four anomaly detection techniques and outlying aspect mining algorithms. Then, we evaluate the performance of anomaly detection techniques and choose the best anomaly detection algorithm. Later, we detect the top *k* anomaly as a query and detect their outlying aspect. Lastly, we evaluate their performance on 16 real-world healthcare datasets. The experimental results show that the latest isolation-based outlying aspect mining measure, SiNNE, has outstanding performance on this task and has promising results.

## Introduction

Despite improvements in healthcare instruments, the presence of medical errors remains a severe challenge [1]. Applying machine learning (ML) and artificial intelligence (AI) algorithms in the healthcare industry helps improve patients’ health more efficiently. According to [2], around 86% of healthcare companies use machine learning and artificial intelligence algorithms. These algorithms help in many ways, such as medical image diagnosis [3, 4], disease detection/classification [5–7], medical data analysis [8], medical data classification [9, 10], drug discovery [8], robot surgery [8], detect anomalous reading [11], etc. Recently, researchers have been interested in detecting abnormal activity in the healthcare industry. Anomaly or outlier^1^ is defined as a data instance that does not conform with the remainder of that set of data instances. In the healthcare domain, an anomaly is referred to as an unusual health condition or activity of a patient [12, 13]. A vast number of applications have been developed to detect anomalies from medical data [14–17]. However, no study has been conducted to find out why these points are considered as an anomaly, i.e., on which set of features a data point is dramatically different than others, as far as we know. The problem of detecting such an explanation leads to outlying aspect mining (a.k.a, outlier explanation, outlier interpretation, outlying subspaces detection). Outlying aspect mining aims to identify the set of features where the given point (or a given anomaly) is most inconsistent with the rest of the data.

**Fig. 1:**
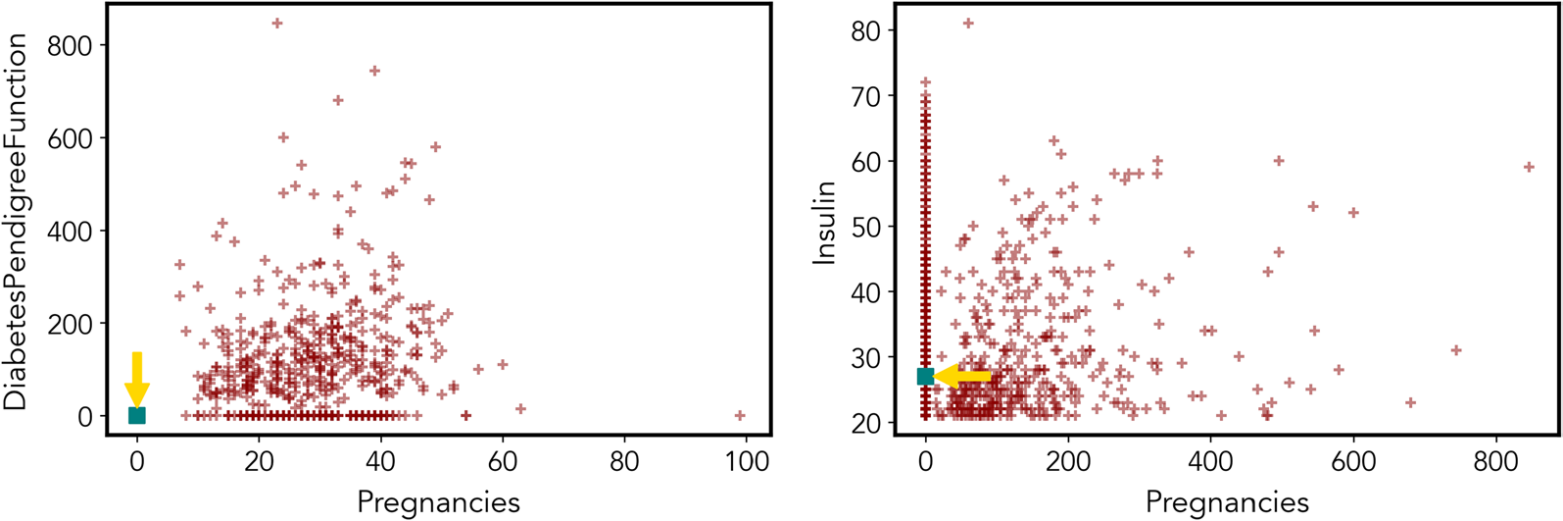
Outlying aspects of Patient A on different features. The square point represents Patient A

In many healthcare applications, a medical officer wants to know the most outlying aspects of a specific patient compared to other patients. For example, you are a doctor having patients with Pima Indian diabetes disease. While treating a particular patient, you want to know in which aspects this patient differs from others. For example, let’s consider the Pima Indian diabetes disease data set.^2^ For ‘Patient A’, the most outlying aspect will be having the highest number of pregnancies and low diabetes pedigree function (see Fig. 1), compared to other subspaces.

Another example is when a medical insurance analyst wants to know in which aspects the given insurance claim is most unusual. The above-given applications are different than anomaly detection. Instead of searching the whole data set for the anomaly, in outlying aspect mining, we are specifically interested in a given data instance. The goal is to find out outlying aspects where a given data instance stands out. Such data instance is called a query $\textbf{q}$.

These interesting applications of outlying aspect mining in the medical domain motivated us to write this paper. In this paper, we first introduce four anomaly detection techniques and outlying aspect mining methods. Later, we evaluate their performance on 16 healthcare datasets. To the best of our knowledge, it is the first time when these algorithms have been applied to healthcare data. Our results have verified their performance on anomaly detection and outlying aspect mining tasks and found that isolation-based algorithm presents promising performance, i.e., iForest perform well in anomaly detection and SiNNE perform well for outlying aspect mining task.

The rest of the paper is organized as follows. Section 2 summarizes the principle and working mechanism of four outlying aspect mining algorithms and anomaly detection algorithms. Next, the experimental setup and results are summarized in Sects. 3 and 4, respectively. Finally, we conclude the paper in Sect. 5.

## Existing methods

Before describing different outlying aspect mining algorithms, we first provide the problem formulation.

### Basic notations and definitions

#### Definition 1

(Problem definition) Given a set of *n* instances ${\mathcal {X}}$ ($\Vert {\mathcal {X}}\Vert$ = *n*) in *d* dimensional space, a data point $\textbf{q} \in {\mathcal {X}}$, is called anomaly iff,

- ${{\textbf {q}}}$ dramatically differs from others in full feature space.

and a subspace *S* is called outlying aspect of $\textbf{q}$ iff,

- outlyingness of $\textbf{q}$ in subspace *S* is higher than other subspaces, and there is no other subspace with the same or higher outlyingness.

**Fig. 2:**
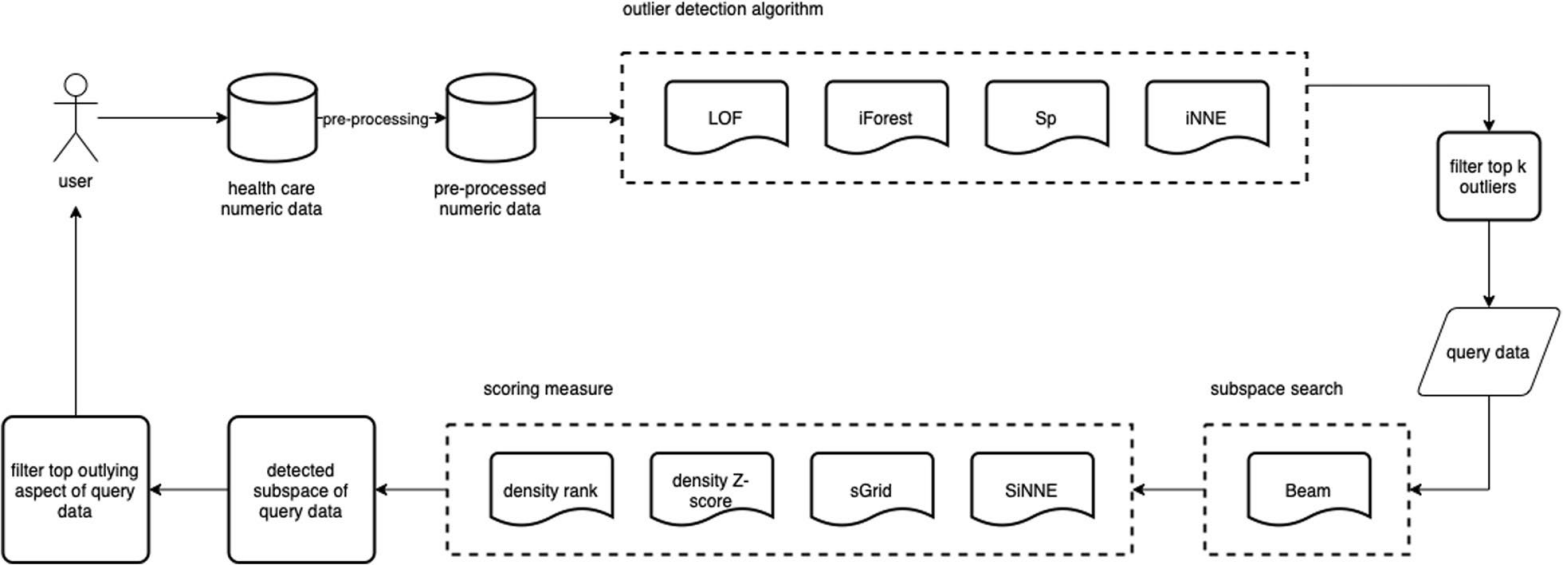
The flowchart

Outlying aspect mining algorithms first require a scoring measure to compute the outlyingness of the query in subspace and a search method to search for the most outlying subspace. In the rest of this section, we review different scoring measures only. For the search part, we will use Beam [18] search method because it is the latest search method and is used in different studies [18–23]. The flowchart of the complete process is presented in Fig. 2.

### Existing anomaly detection scoring measures

#### LOF

The core idea of density-based anomaly detection is the density of the anomalous object is significantly different from the normal instance. The first local density-based approach, called LOF, which stands for **L**ocal **O**utlier **F**actor introduced by [24], which is the widely used local outlier detection approach. For any data object, the LOF score is the ratio of the average local density of its *k*-nearest neighbors to its local density [25]. The LOF score of data object ${{\textbf {q}}}$ is defined as follows:

$$\begin{aligned} \text{ LOF }({{\textbf {q}}}) = \frac{\sum \limits _{x \in N^k({{\textbf {q}}})} lrd(x)}{\Vert N^k({{\textbf {q}}})\Vert \times lrd({{\textbf {q}}})} \end{aligned}$$

where $lrd({{\textbf {q}}}) = \frac{\Vert N^k ({{\textbf {q}}})\Vert }{\sum \limits _{x \in N^k({{\textbf {q}}})} max(dist^k(x,D),dist({{\textbf {q}}},x))}$, $N^k({{\textbf {q}}})$ is a set of *k*-nearest neighbours of ${{\textbf {q}}}$, $dist({{\textbf {q}}},x)$ is a distance between ${{\textbf {q}}}$ and *x* and $dist^k({{\textbf {q}}},D)$ is the distance between ${{\textbf {q}}}$ and its *k*-NN in ${\mathcal {X}}$. The LOF score represents the sparseness of the data object. Data objects with higher LOF values are considered as anomalies.

#### iForest

Liu et al. [26] presented a framework called **I**solation **Forest** or iForest, which isolates each data point by axis-parallel partitioning of the attribute space. To the best of our knowledge, iForest is the first technique that uses an isolation mechanism to detect anomalies.

iForest builds an ensemble of trees called isolation trees (iTree). Each iTree is built using a randomly selected sub-sample without replacement from the data set. A random split is performed at each node on a randomly selected point from attribute space. The partition will terminate once all the nodes have only one data object or nodes reach the tree’s height limit for iTree. The anomaly score for ${{\textbf {q}}} \in {\mathcal {R}}^d$ based on iForest is defined as:

$$\begin{aligned} \text{ iForest }({{\textbf {q}}}) = \frac{1}{t} \sum \limits _{i=1}^t l_i({{\textbf {q}}}) \end{aligned}$$

where $l_i({{\textbf {q}}})$ is the path length of ${{\textbf {q}}}$ in tree $T_i$.

#### Sp

Rather than searching for *k*-nearest neighbor in the data set, [27] employs scoring measure based on the nearest neighbor (*k* =1) in random sub-samples (${\mathcal {S}} \subset D$). The Sp score of data object ${{\textbf {q}}}$ is defined as follows:

$$\begin{aligned} \text{ Sp }({{\textbf {q}}}) = \min \limits _{x \in {\mathcal {S}}} dist({{\textbf {q}}},x) \end{aligned}$$

where $dist({{\textbf {q}}},x)$ is a distance between ${{\textbf {q}}}$ and *x*.

In [27], authors have shown that Sp performs better than state-of-the-art anomaly detector LOF and runs faster than LOF.

#### iNNE

Bandaragoda et al. [28] proposed iNNE, which is stands for **i**solation using **N**earest **N**eighbor **E**nsemble. The core idea behind iNNE is an anomaly is far away from its nearest neighbor, and the inverse is true for the regular object. iNNE implementation is influenced by iForest and LOF. The critical difference between iNNE and iForest is that iForest builds a tree from subspaces while iNNE builds hyperspheres using all dimensions. An isolation score of ${{\textbf {q}}}$ is defined as follows:

$$\begin{aligned} I({{\textbf {q}}}) = {\left\{ \begin{array}{ll} 1 - \frac{\tau (\eta _{cnn({{\textbf {q}}})})}{\tau (cnn({{\textbf {q}}}))}, &{}if {{\textbf {q}}} \in \bigcup _{c \in {\mathcal {S}}} B(c),\\ \quad \quad \quad \quad 1, &{}otherwise \end{array}\right. } \end{aligned}$$

where $cnn({{\textbf {q}}}) = \displaystyle \mathop {\mathrm {arg\,min}}\limits _{c \in S} \{ \tau (c): {{\textbf {q}}} \in {\mathcal {B}}(c) \}$, ${\mathcal {S}}$ is set of randomly selected sub-samples, $\Vert {\mathcal {S}}\Vert = \psi$, ${\mathcal {B}}(c)$ is a hypersphere centered at *c* with radius $\tau (c) = || c - \eta _c ||$, where $\eta _c$ is nearest neighbour of *c*. The anomaly score for data object ${{\textbf {q}}}$ is defined as:

$$\begin{aligned} \text{ iNNE }({{\textbf {q}}}) = \frac{1}{t} \sum \limits _{i=1}^t I_i({{\textbf {q}}}) \end{aligned}$$

where $I_i({{\textbf {q}}})$ is isolation score based on sub-sample in $i^{th}$ set.

### Outlying aspect mining algorithms

#### OAMiner

Duan et al. [29] introduce **O**utlying **A**spect **Miner** (**OAMiner** in short), which uses a Kernel Density Estimation (KDE) [30] based scoring measure to compute the outlyingness of query $\textbf{q}$ in subspace *S*:

$$\begin{aligned} f_{S}(\textbf{q}) = \frac{1}{n(2 \pi )^{\frac{m}{2}} \prod \limits _{i \in S} h_{i}} \sum \limits _{\textbf{x} \in {\mathcal {O}}} e^ {- \sum \limits _{i\in S} \frac{(q.i - x.i)^2}{2 h^2_{i}}} \end{aligned}$$

where $f_S(\textbf{q})$ is a kernel density estimation of $\textbf{q}$ in subspace *S*, *m* is the dimensionality of subspace *S* ($|S|=m$), $h_{i}$ is the kernel bandwidth in dimension *i*.

Duan et al. [29] have stated that density is a bias towards high-dimensional subspaces—density tends to decrease as the dimension increases. Thus, to remove the effect of dimensionality bias, they proposed using the query’s density rank as a measure of outlyingness. To find the most outlying subspace of the query, the density of all data points needs to compute in each subspace, where the subspace with the best rank is selected as an outlying aspect of the given query.

OAMiner systematically enumerates all the possible subspaces. In OAMiner, the author has used the set enumeration tree approach [31], which is widely used by the data mining research community. OAMiner searches for subspaces by traversing a depth-first manner [32]. OAMiner used some anti-monotonicity properties to prune the subspaces. Given data set ${\mathcal {O}}$, a query object $\textbf{q}$ and subspace *S*, if $rank(f_{S}(\textbf{q}))$ = 1, then every super-set of *S* cannot be a minimal subspace and thus can be pruned.

#### Beam

Vinh et al. [18] captures the concept of dimensionality unbiasedness and further investigates dimensionally unbiased scoring functions. Dimensionality unbiasedness is an essential property for outlying measures because the query object is compared in different subspaces with a different number of dimensions. They proposed two novel outlying scoring metrics (1) density *Z*-score and (2) **i**solation **Path** score (iPath in short). Their work showed that the proposed *Z*-score and iPath are dimensionally unbiased.

Therein, the density *Z*-score is defined as follows:

$$\begin{aligned} Z\hbox {-Score} ({\tilde{f}}_S(\textbf{q})) \triangleq \frac{{\tilde{f}}_S(\textbf{q}) -\mu _{{\tilde{f}}_S}}{\sigma _{{\tilde{f}}_S}} \end{aligned}$$

where $\mu _{f_S}$ and $\sigma _{f_S}$ are the mean and standard deviation of the density of all data instances in subspace *S*, respectively.

The iPath score is motivated by **i**solation **Forest** (iForest) anomaly detection approach [26]. The intuition behind iForest is that anomalies are few and susceptible to isolation. iForest constructs *t* trees, where each tree is built from randomly selected sub-samples $\psi$ ($\psi \ll n$). Later, it divides using the axis-parallel random splits. Since in the outlying aspect mining context, the main focus is on the path length of the query; thus, authors have ignored other parts of the tree. In outlying aspect mining, the intuition behind the iPath score is that in the most outlying subspace, a given query is easy to isolate than the rest of the data.

The process of calculating the iPath of query $\textbf{q}$ w.r.t. sub-samples $\psi$ of the data is

$$\begin{aligned} iPath_S(\textbf{q}) = \frac{1}{t} \sum \limits _{i=1}^t l_S^i(\textbf{q}) \end{aligned}$$

where $l_S^i(\textbf{q})$ is path length of $\textbf{q}$ in $i^{th}$ tree and subspace *S*.

Vinh et al. [18] was the first to coin the term dimensionality unbiasedness.

##### Definition 2

(*Dimensionality unbiased* [18]) A dimensionality unbiased outlyingness measure (*OM*) is a measure of which the baseline value, i.e., average value for any data sample ${\mathcal {O}} = \{o_1, o_2, \cdots , o_n \}$ drawn from a uniform distribution, is a quantity independent of the dimension of the subspace *S*, i.e.,

$$\begin{aligned} E[OM_S(x)\Vert x \in {\mathcal {O}}] = \frac{1}{n} \sum \limits _{x \in {\mathcal {O}}} OM(x) = \text{ const. } \text{ w.r.t } \Vert S\Vert \end{aligned}$$

In [18, Theorem 3], it is proven that rank transformation and *Z*-score normalization have resulted in a constant average value in any data distribution. Furthermore, it is worth noting that the *Z*-score scoring function is not only normalized but also the variance of the normalized measures that are constant to dimensions.

The overall beam search process is divided into three stages. All 1-D subspaces are inspected in the first stage to identify trivial outlying features. In the subsequent stage, an exhaustive search is performed on all possible 2 dimensional subspaces. In the third stage, the beam search is implemented at level *l*. The beam algorithm only keeps top *W* subspaces (called beam width) in the search process. The total number of subspace considered by the beam algorithm is in the order of $O(d^2 + W \ \ d_{max})$ where $d_{max}$ is the maximum dimension of subspace, and *W* is the beam width.

#### sGrid

Wells and Ting [23] introduced a simple grid-based density estimator called sGrid. sGrid is a smoothed variant of a grid-based density estimator [30]. Let ${\mathcal {O}}$ be a collection of *n* data objects in *D*-dimensional space, *x*.*S* be a projection of a data object $x \in {\mathcal {O}}$ in subspace *S*. The sGrid density of point $\textbf{q}$ is computed as points that fall in a bin that covers point $\textbf{q}$ and its surrounding neighbors.

Their work showed that the proposed density estimator has advantages over the existing kernel density estimator in outlying aspect mining by replacing the kernel density estimator with sGrid. By replacing KDE with the sGrid density estimator, OAMiner [29] and Beam [18] run two orders of magnitude faster than their original implementation. However, sGrid is not a dimensionally unbiased measure, requiring *Z*-Score normalization. Again, it makes sGrid computationally inefficient.

#### SiNNE

Very recently, [21] proposed a **S**imple **I**solation score using **N**earest **N**eighbor **E**nsemble (SiNNE in short) measure which from Isolation using Nearest Neighbor Ensembles (iNNE in short) method for anomaly detection [28]. SiNNE constructs *t* ensemble of models (${\mathcal {M}}_1, {\mathcal {M}}_2, \cdots , {\mathcal {M}}_t$). Each model ${\mathcal {M}}_i$ is constructed from randomly chosen sub-samples (${\mathcal {D}}_i \subset {\mathcal {O}}, \Vert {\mathcal {D}}_i\Vert = \psi < n)$. Each model has $\psi$ hyperspheres, where a radius of the hypersphere is the euclidean distance between *a* ($a \in {\mathcal {D}}_i)$ to its nearest neighbor in ${\mathcal {D}}_i$.

The outlying score of $\textbf{q}$ in model ${\mathcal {M}}_i$, $I(q\Vert {\mathcal {M}}_i) = 0$ if $\textbf{q}$ falls in any of the ball and 1 otherwise. The final outlying score of $\textbf{q}$ using *t* models is:

$$\begin{aligned} \text{ SiNNE }({{\textbf {q}}}) = \frac{1}{t} \sum \limits _{i=1}^t I({{\textbf {q}}}\Vert {\mathcal {M}}_i) \end{aligned}$$

In their work, they argue that *Z*-score normalization is biased towards a subspace having high-density variance, and the definition of dimensionality unbiasedness needs to be revised. Furthermore, SiNNE is computationally faster than density and distance-based measures.

## Experimental setup

### Datasets

In this study, we used 16 publicly available benchmarking medical datasets for anomaly detection; *BreastW* and *Pima* are from [33],^3^*Annthyroid*, *Cardiotocography*, *Heart disease*, *Hepatitis*, *WDBC* and *WPBC* are from [34]^4^ and *Arrhythmia*, *Lympho*, *Mammography*, *Musk*, *Thyroid*, *Vertebral*, *WBC*, and *Yeast* are from [35].^5^ The summary of each data set is provided in Table 1.

**Table 1:** Characteristics of datasets used

Data set	#datasize (N)	#dimension (d)	#anomalies
Annthyroid	7129	21	534
Arrhythmia	452	274	66
BreastW	683	9	239
Cardiotocography	2114	21	176
Diabetes	768	8	268
Heart disease	270	13	10
Hepatitis	80	19	13
Lympho	148	18	6
Mammography	11183	6	260
Musk	3062	166	97
Pima	768	8	268
Thyroid	3772	6	93
Vertebral	240	6	30
WBC	278	30	21
WDBC	367	30	10
WPBC	198	33	47

### Algorithm implementation and parameters

We use PyOD [36] Python library to implement anomaly detection algorithms. In terms of implementation of OAM algorithms, we used Java implementation of sGrid and SiNNE, which is made available by the authors [23] and [21], respectively. We implemented RBeam and Beam in Java using WEKA [37].

We used the default parameters of each algorithm as suggested in respective papers unless specified otherwise.

***Anomaly detection algorithm:***

- LOF: the size of nearest neighbor (*k*) = 10;
- iForest: number of sets *t*=100, and sub-sample size $\psi$=256;
- Sp: sub-sample size $\psi$=20; and
- iNNE: number of sets *t*=100, and sub-sample size $\psi$=8.

***Outlying aspect mining algorithms:***

- Density rank and Density *Z*-score: KDE use Gaussian kernel with default bandwidth as suggested by [38];
- sGrid: block size parameter *w* = 64;
- SiNNE: sub-sample size $\psi$ = 8, and ensemble size *t* = 100; and
- Beam search: beam width *W* = 100, and maximum dimensionality of subspace $\ell$ = 3.

### Evaluation measure

We used the area under the ROC curve (AUC) [39] and precision at n (*P*@*n*)^6^ [40] as a measure of effectiveness for anomaly ranking produced by an anomaly detector. An anomaly detector with a high AUC indicates better detection accuracy, whereas a low AUC indicates low detection accuracy.

Samariya and Ma [20] proposed a new kernel mean embedding-based evaluation measure in the outlying aspect mining domain. The intuition behind the evaluation measure is that in most outlying aspects, a query $\textbf{q}$ is far from the distribution of data in those aspects.

#### Definition 3

The quality of discovered aspects (or subspace(s)) ${\mathcal {S}}$ for a query $\textbf{q}$ is computed as

1 $$\begin{aligned} f_{\mathcal {S}}(\textbf{q}, {\mathcal {X}}) = \frac{1}{n} \sum \limits _{x \in {\mathcal {X}}} K_{\mathcal {S}} (\textbf{q}, x) \end{aligned}$$

where $K_{\mathcal {S}} (\textbf{q}, x)$ is a kernel similarity between $\textbf{q}$ and *x* in subspace ${\mathcal {S}}$.

Therein, authors used chi-square kernel [41], computed as follows.

$$\begin{aligned} K_{\mathcal {S}} (\textbf{q}, x) = 1 - \sum \limits _{i \in {\mathcal {S}}} 2 \frac{(\textbf{q}_i - x_i)^2}{(\textbf{q}_i + x_i)} \end{aligned}$$

All experiments were conducted on a machine with an Intel 8-core i9 CPU and 16 GB main memory, running on macOS Big Sur version 11.1. We run each job on multiple single CPU treads, which is done using GNU parallel [42].

**Table 2:** AUC scores of LOF, iForest, Sp, and iNNE anomaly detection methods on 16 real-world healthcare datasets

Dataset	AUC				Runtime(seconds)			
	LOF	iForest	Sp	iNNE	LOF	iForest	Sp	iNNE
*annthyroid*	0.64	**0.68**	0.52	0.56	0.10	0.38	0.01	0.50
*arrhythmia*	0.82	** 0.83**	0.78	0.74	0.01	0.24	0.01	0.18
*breastw*	0.62	**1.00**	0.99	0.65	0.01	0.21	<0.01	0.13
*cardiotocography*	0.56	0.68	0.63	**0.74**	0.02	0.25	<0.01	0.13
*diabetes*	0.58	**0.66**	0.58	**0.66**	0.01	0.21	<0.01	0.10
*heart*_*disease*	0.41	**0.71**	0.52	0.53	0.01	0.19	<0.01	0.10
*hepatitis*	**0.77**	0.74	0.67	0.30	0.01	0.18	<0.01	0.10
*lympho*	**1.00**	**1.00**	0.95	0.99	0.01	0.18	<0.01	0.09
*mammography*	0.73	**0.86**	0.77	0.78	0.21	0.45	0.21	0.67
*musk*	0.39	**1.00**	0.03	**1.00**	0.04	0.43	0.02	0.39
*pima*	0.58	**0.73**	0.59	0.61	0.01	0.21	<0.01	0.13
*thyroid*	0.83	** 0.97**	0.95	0.95	0.08	0.28	<0.01	0.26
*vertebral*	**0.59**	0.49	0.37	0.40	0.01	0.18	<0.01	0.09
*wbc*	**0.95**	0.94	0.92	0.83	0.01	0.19	<0.01	0.11
*wdbc*	**1.00**	0.90	0.78	0.76	0.01	0.19	<0.01	0.11
*wpbc*	0.46	0.30	**0.51**	**0.51**	0.01	0.18	<0.01	0.10
Avg. AUC	0.68	0.78	0.66	0.69	–	–	–	–

Best AUC results are indicated in bold

## Empirical evaluation

In this section, we present the result of four anomaly detection methods; LOF, iForest, Sp, and iNNE and four outlying scoring measures; Kernel Density Rank (RBeam), Density *Z*-score (Beam), sGrid *Z*-score (sBeam) and SiNNE (SiBeam) using Beam search on medical datasets. All experiments were run for 1 h, and unfinished tasks were killed and presented as ‘$\ddagger$’.

### Experiment-1: Performance of anomaly detection algorithms

In this sub-section, we presented the results of four anomaly detection techniques: LOF, iForest, Sp, and iNNE in terms of AUC.

The AUC comparison of LOF, iForest, Sp, and iNNE is presented in Table 2 (c.f. columns 2 to 5 of Table 2). It is interesting to note that no specific anomaly detection algorithm performs best in each dataset. However, iForest is the best-performing measure with having the best AUC in 10 datasets. In the last row of Table 2, the Avg. AUC of each anomaly detection method shows that iForest produced the best AUC while Sp had a significantly low AUC. Whereas LOF and iNNE produce comparative results.

The total runtime, which includes pre-processing, model building, ranking *n* instances, and computing AUC, is presented in Table 2 (c.f. columns 6 to 9 of Table 2). Overall, Sp is the fastest measure compared to others. While iForest and iNNE almost take similar time.

### Experiment-2: Performance of outlying aspect mining algorithms

**Table 3:** Comparison of outlying aspects discovered by RBeam, Beam, sGBeam, and SiBeam on ***annthyroid***, ***arrhythmia***, ***breastw*** and ***cardiotocography*** datasets

	${{\textbf {q}}}$-id	RBeam	Beam	sGBeam	SiBeam
*annthyroid*	952	$\ddagger$	$\ddagger$	{17} (0.89)	{2, 5, 17} (− 2.77)
	1053			{14} (− 1.00)	{2, 14} (− 1.26)
	1204			{18} (0.84)	{0, 18, 19} (0.41)
	2286			{12} (− 0.98)	{3, 12, 20} (− 1.03)
	3305			{11} (− 0.97)	{9, 11, 19} (− 3.07)
	3337			{7} (− 0.97)	{3, 7, 17} (− 2.96)
	3606			{12} (− 0.98)	{0, 3, 12} (− 3.04)
	4921			{20} (0.09)	{1, 18} (− 0.77)
	5397			{7} (− 0.97)	{7, 8, 16} (− 3.14)
	6871			{11} (− 0.97)	{0, 11, 13} (− 1.37)
*arrhythmia*	85	{0, 15} (− 0.03)	{220} (0.79)	{14, 258} (− 0.99)	{26, 133} (− 0.99)
	141	{0, 1} (− 0.95)	{220} (0.79)	{14, 69} (− 1.00)	{2, 69} (− 2.52)
	210	{0, 186} (− 0.44)	{220} (0.89)	{14, 89} (− 0.97)	{53, 181, 196} (0.46)
	297	{0, 35} (− 0.18)	{51} (0.14)	{14, 113} (− 0.99)	{0, 16, 83} (− 1.18)
	308	{36, 164, 166} (0.32)	{220} (0.89)	{14, 16} (− 1.00)	{3, 16, 19} (− 1.16)
	316	{0} (− 0.12)	{220} (0.84)	{2} (− 0.04)	{2, 93, 246} (− 0.33)
	379	{0, 24} (− 0.74)	{47} (− 0.12)	{14, 239} (− 1.00)	{228, 257} (− 0.97)
	403	{0, 9} (− 0.92)	{220} (0.85)	{14, 150} (− 0.99)	{53, 214} (0.50)
	424	{0, 11} (− 0.14)	{51} (0.02)	{14, 223} (0.49)	{100, 108, 204} (0.12)
	449	{0, 167} (− 0.94)	{220} (0.77)	{14, 219} (− 0.99)	{65, 257} (− 0.80)
*breastw*	8	{0, 8} (− 0.22)	{6} (0.46)	{8} (0.23)	{3, 6, 8} (− 0.72)
	70	{0, 6} (− 0.37)	{0, 6} (− 0.37)	{6} (0.39)	{0, 5, 6} (− 0.94)
	108	{0, 6, 7} (− 0.56)	{6} (0.64)	{6} (0.64)	{0, 5, 6} (− 0.62)
	127	{0, 4} (− 0.87)	{4} (− 0.10)	{4} (− 0.10)	{0, 4, 7} (− 1.29)
	158	{5} (0.50)	{5} (0.50)	{8} (− 0.17)	{0, 3, 8} (− 1.28)
	161	{2, 5, 7} (− 1.27)	{0} (0.16)	{8} (− 0.79)	{5, 7, 8} (− 1.77)
	286	{0, 2} (− 0.06)	{0} (0.43)	{4} (0.68)	{2, 4, 6} (− 0.36)
	305	{0, 1, 4} (− 0.57)	{0, 4} (− 0.09)	{4} (0.68)	{0, 1, 4} (− 0.57)
	333	{4, 7} (0.07)	{0, 7} (− 0.20)	{8} (0.87)	{4, 6, 7} (− 0.48)
	673	{6} (0.46)	{6} (0.46)	{8} (− 0.37)	{0, 4, 8} (− 1.34)
*cardiotocography*	140	{0, 14} (0.30)	{0} (0.71)	{15}(− 0.84)	{0, 15} (− 1.13)
	165	{1, 7} (− 0.41)	{5} (0.55)	{7}(− 0.27)	{0, 7} (− 0.34)
	383	{1, 12, 16} (0.00)	{16} (0.78)	{15}(0.32)	{3, 16, 19} (− 0.43)
	432	{5, 6} (0.48)	{11} (0.52)	{15} (− 0.45)	{2, 15} (− 0.48)
	784	{0, 3} (0.00)	{3} (0.17)	{3} (0.17)	{3, 6, 7} (− 0.42)
	1141	{1, 3, 8} (− 0.53)	{4, 20} (0.10)	{8} (− 0.03)	{4, 8, 20} (− 0.94)
	1476	{16, 19, 20} (− 0.02)	{3} (0.70)	{9} (− 0.99)	{9, 14} (− 1.13)
	1477	{3, 16, 20} (0.29)	{16} (0.78)	{9} (− 0.99)	{3, 4, 9} (− 1.36)
	1635	{0, 1} (− 0.62)	{3} (0.45)	{3} (0.45)	{1, 20} (− 0.76)
	2074	{3} (− 0.29)	{3} (− 0.29)	{3} (− 0.29)	{1, 3} (− 0.50)

${{\textbf {q}}}$-id represents query point index; the numbers in the bracket are attribute indices (subspace); the numbers in the parenthesis are quality of subspace (lower the better)

Best results are underlined

**Table 4:** Comparison of outlying aspects discovered by RBeam, Beam, sGBeam, and SiBeam on ***diabetes***, ***ecoli***, ***heart***_***disease*** and ***hepatitis***

	${{\textbf {q}}}$-id	RBeam	Beam	sGBeam	SiBeam
*diabetes*	9	{5, 7} (− 77.23)	{5} (− 62.99)	{5} (− 62.99)	{2, 5, 7} (− 94.57)
	13	{0, 4} (− 1350.19)	{1} (− 39.00)	{4} (− 1345.59)	{4, 5, 7} (− 1366.80)
	106	{0, 2} (− 41.26)	{2} (− 36.66)	{2} (− 36.66)	{1, 2, 6} (− 50.68)
	125	{2, 5} (− 50.97)	{5} (− 13.69)	{5} (− 13.69)	{2, 5} (− 50.97)
	145	{3, 5, 6} (− 80.16)	{5} (− 62.99)	{5} (− 62.99)	{0, 2, 5} (− 79.69)
	177	{0, 5} (− 34.06)	{5} (− 26.37)	{5} (− 26.37)	{0, 5} (− 34.06)
	362	{2, 7} (− 49.59)	{2} (− 24.59)	{7} (− 24.00)	{2, 3} (− 50.67)
	459	{0, 7} (− 50.69)	{7} (− 43.38)	{7} (− 43.38)	{3, 7} (− 66.01)
	579	{0, 3} (− 117.26)	{3} (− 113.96)	{3} (− 113.96)	{0, 3} (− 117.26)
	672	{1, 2} (− 59.06)	{2} (− 23.07)	{2} (− 23.07)	{0, 1, 2} (− 67.65)
*heart*_*disease*	1	{0, 4} (− 0.04)	{1, 12} (− 1.49)	{4} (0.15)	{3, 4} (0.00)
	13	{2} (− 0.45)	{2, 6, 8} (− 2.13)	{2, 6, 8} (− 2.13)	{2, 7, 11} (− 1.36)
	48	{2, 11} (− 0.78)	{12} (0.13)	{12} (0.13)	{2, 11, 12} (− 1.65)
	98	{1, 10, 12} (− 2.07)	{1, 12} (− 1.49)	{1, 12} (− 1.49)	{1, 7, 12} (− 1.57)
	101	{0, 7} (− 0.39)	{7} (− 0.20)	{7} (− 0.20)	{7, 9, 11} (− 0.89)
	103	{11} (− 0.34)	{3} (0.70)	{5} (− 0.70)	{1, 3, 11} (− 1.28)
	118	{1, 2, 7} (− 2.00)	{10} (− 0.11)	{10} (− 0.11)	{2, 6, 10} (− 1.92)
	175	{1, 6, 11} (− 2.72)	{1, 7} (− 0.65)	{5, 6} (− 1.73)	{1, 5, 7} (− 2.35)
	243	{9, 10, 11} (− 1.67)	{10} (− 0.11)	{10} (− 0.11)	{8, 9, 10} (− 1.48)
	256	{6, 8} (− 1.36)	{2, 8} (− 0.54)	{5, 6} (− 1.73)	{0, 5, 8} (− 2.16)
*hepatitis*	4	{8, 16} (− 0.31)	{4, 16} (0.04)	{13} (0.71)	{5, 13, 16} (− 0.32)
	5	{5} (− 0.23)	{2, 13} (− 0.29)	{2, 6, 8} (− 2.70)	{11, 14, 18} (− 1.68)
	18	{2, 14} (− 0.94)	{14} (0.11)	{14} (0.11)	{9, 14, 18} (− 1.09)
	27	{0, 8, 17} (− 0.53)	{0, 8, 17} (− 0.53)	{2, 4, 5} (− 1.53)	{0, 6, 17} (− 0.12)
	29	{2, 8, 17} (− 1.17)	{2, 4, 8} (− 2.30)	{4, 8, 9} (− 2.88)	{0, 4, 9} (− 2.06)
	34	{2, 3, 4} (− 2.73)	{2, 3, 4} (− 2.73)	{2, 3, 4} (− 2.73)	{3, 4, 17} (− 1.88)
	39	{4, 16} (− 0.50)	{4, 16} (− 0.50)	{4, 16} (− 0.50)	{4, 16} (− 0.50)
	40	{2, 5, 8} (− 2.13)	{2, 5} (− 1.18)	{0, 4, 18} (− 0.71)	{8, 16, 18} (− 0.88)
	59	{3, 4, 18} (− 2.95)	{3, 18} (− 1.65)	{3, 18} (− 1.65)	{0, 10, 15} (− 0.40)
	63	{0, 2, 10} (− 2.22)	{0, 2, 4} (− 1.54)	{2, 6, 8} (− 2.70)	{0, 7, 17} (− 0.53)
*lympho*	2	{1, 9} (− 0.69)	{10} (− 0.23)	{8} (− 0.93)	{3, 8, 14} (− 2.80)
	3	{5, 7} (0.20)	{10} (− 0.23)	{8} (− 0.93)	{1, 8, 13} (− 2.24)
	29	{9, 10} (− 0.68)	{10, 14} (− 0.52)	{5, 8} (0.01)	{2, 8, 17} (− 1.73)
	34	{1, 11, 15} (− 0.34)	{7, 9} (0.12)	{11, 16} (− 0.13)	{4, 11, 16} (− 0.63)
	47	{7, 9} (− 0.52)	{7, 9} (− 0.52)	{7, 9} (− 0.52)	{7, 9, 16} (− 1.04)
	53	{1, 12, 13} (− 3.30)	{5, 12, 14} (− 0.33)	{1, 2, 16} (− 0.37)	{2, 12, 13} (− 3.55)
	64	{11, 14, 15} (− 1.28)	{10, 14} (− 0.52)	{10, 14} (− 0.52)	{11, 14, 16} (− 1.36)
	95	{5, 17} (− 6.18)	{14, 15, 17} (− 6.78)	{5, 8} (0.01)	{9, 15, 17} (− 7.67)
	113	{9, 12, 13} (− 4.05)	{11, 13} (− 2.94)	{11, 13} (− 2.94)	{9, 12, 13} (− 4.05)
	127	{0, 12} (− 1.55)	{7, 12} (− 1.00)	{7, 12} (− 1.00)	{0, 2, 12} (− 2.10)

${{\textbf {q}}}$-id represents query point index; the numbers in the bracket (subspace) are attribute indices

Best results are underlined

**Table 5:** Comparison of outlying aspects discovered by RBeam, Beam, sGBeam, and SiBeam on ***lympho***, ***musk***, ***pima*** and ***thyroid***

	${{\textbf {q}}}$-id	RBeam	Beam	sGBeam	SiBeam
*mammography*	539	{0, 1} (0.12)	{0} (0.68)	{4} (− 0.92)	{0, 4} (− 1.24)
	2774	{3, 5} (− 0.50)	{0} (0.50)	{0} (0.50)	{0, 4} (0.10)
	4653	{2} (0.94)	{2} (0.94)	{5} (0.33)	{1, 2} (0.79)
	5024	{3, 5} (− 1.11)	{0} (0.78)	{3} (− 0.58)	{1, 3} (− 0.86)
	5350	{2} (− 0.15)	{2} (− 0.15)	{2} (− 0.15)	{2, 3} (− 0.31)
	6104	{0} (− 0.86)	{0} (− 0.86)	{0} (− 0.86)	{0} (− 0.86)
	7096	{2} (− 0.27)	{2} (− 0.27)	{2} (− 0.27)	{0, 2} (− 0.29)
	8746	{2} (− 0.31)	{2} (− 0.31)	{2} (− 0.31)	{0, 2} (− 0.34)
	10144	{2} (− 0.89)	{2} (− 0.89)	{2} (− 0.89)	{0, 1, 2} (− 1.06)
	10606	{3} (− 0.61)	{0} (0.67)	{3} (− 0.61)	{3} (− 0.61)
*musk*	348	$\ddagger$	$\ddagger$	{146} (1239.49)	{138, 146, 162} (1350.06)
	831			{112} (161.63)	{77, 115, 150} (− 207.01)
	845			{112} (161.63)	{77, 93, 115} (− 224.55)
	866			{65, 150} (32.41)	{17, 41, 135} (− 407.11)
	1270			{47, 107} (327.25)	{30, 82, 143} (1621.84)
	1283			{77, 88} (64.86)	{50, 140, 157} (2108.53)
	1287			{113} (43.94)	{49, 82, 100} (2153.20)
	2016			{47, 80} (617.47)	{98, 101, 111} (151.13)
	2827			{34, 91, 155} (559.94)	{69, 161, 163} (− 484.13)
	3054			{129, 130} (− 2002.80)	{123, 149, 161} (1471.68)
*pima*	5	{2, 5} (0.48)	{5} (0.78)	{5} (0.78)	{2, 3, 5} (0.16)
	14	{0, 5} (0.14)	{5} (0.59)	{5} (0.59)	{1, 5, 6} (0.44)
	26	{0, 7} (− 0.64)	{7} (− 0.21)	{7} (− 0.21)	{2, 3, 7} (− 0.51)
	36	{4, 6} (− 1.57)	{6} (− 0.20)	{4} (− 0.37)	{4, 6} (− 1.57)
	124	{0, 2} (0.42)	{2} (0.69)	{2} (0.69)	{2, 5} (0.61)
	304	{0, 3} (− 0.36)	{3} (− 0.16)	{3} (− 0.16)	{0, 3} (− 0.36)
	306	{2, 7} (− 1.09)	{7} (0.04)	{2} (− 0.13)	{1, 2, 7} (− 1.14)
	410	{0, 4} (− 0.86)	{1} (0.80)	{4} (− 0.59)	{0, 4, 7} (− 1.49)
	430	{3, 5, 6} (− 0.24)	{5} (0.05)	{5} (0.05)	{0, 2, 5} (− 0.48)
	695	{5, 6} (− 0.71)	{5} (0.05)	{5} (0.05)	{5, 6} (− 0.71)
*thyroid*	38	{0, 5} (− 0.28)	{1} (0.97)	{5} (− 0.16)	{0, 5} (− 0.28)
	704	{2, 3} (0.04)	{3} (0.07)	{5} (0.45)	{1, 5} (0.44)
	1344	{2} (0.75)	{3} (0.79)	{2} (0.75)	{0, 2, 3} (0.43)
	1376	{0, 2} (0.40)	{3} (0.72)	{2} (0.75)	{2, 3} (0.47)
	1881	{2} (− 0.14)	{3} (0.77)	{5} (0.57)	{2} (− 0.14)
	2503	{1} (− 0.96)	{1} (− 0.96)	{1} (− 0.96)	{1, 3} (− 1.35)
	2527	{3, 4, 5} (0.84)	{3} (0.89)	{5} (0.97)	{3, 4, 5} (0.84)
	2548	{4} (0.68)	{0} (0.58)	{4} (0.68)	{0, 2, 4} (0.07)
	2906	{0, 4} (0.26)	{0} (0.71)	{2} (0.79)	{0, 4} (0.26)
	2931	{1} (− 0.47)	{1} (− 0.47)	{1} (− 0.47)	{1, 4, 5} (− 0.80)

${{\textbf {q}}}$-id represents query point index; the numbers in the bracket (subspace) are attribute indices

Best results are underlined

**Table 6:** Comparison of outlying aspects discovered by RBeam, Beam, sGBeam, and SiBeam on ***vertebral***, ***wbc***, ***wdbc*** and ***wpbc***

	${{\textbf {q}}}$-id	RBeam	Beam	sGBeam	SiBeam
*vertebral*	9	{4, 5} (− 74.48)	{4} (− 10.37)	{4} (− 10.37)	{0, 4} (− 27.91)
	51	{1, 2} (− 44.30)	{0, 2} (− 27.48)	{1, 5} (− 149.75)	{1, 2} (− 44.30)
	52	{0, 1, 3} (− 38.05)	{3} (− 19.26)	{3} (− 19.26)	{0, 1} (− 17.79)
	112	{1} (− 217.58)	{1} (− 217.58)	{1} (− 217.58)	{0, 1, 2} (− 240.19)
	115	{0} (− 50.18)	{0} (− 50.18)	{5} (− 673.90)	{2, 3} (− 82.33)
	118	{0, 2, 4} (− 16.63)	{0, 4} (− 8.96)	{4} (− 4.09)	{1, 4, 5} (− 51.73)
	134	{0, 2} (− 21.43)	{0, 2} (− 21.43)	{0, 2} (− 21.43)	{0, 1, 2} (− 27.65)
	167	{4} (− 22.75)	{4} (− 22.75)	{4} (− 22.75)	{3, 4, 5} (− 78.99)
	197	{0, 2} (− 68.02)	{2} (− 63.13)	{5} (− 126.66)	{2, 4} (− 67.97)
	237	{1} (− 61.31)	{1} (− 61.31)	{1} (− 61.31)	{1, 5} (− 95.51)
*wbc*	22	{14, 21} (0.22)	{4} (0.75)	{4} (0.75)	{8, 21, 29} (0.11)
	56	{11, 24, 28} (0.29)	{28} (0.68)	{11} (0.67)	{4, 18, 28} (0.50)
	69	{0, 17} (− 0.25)	{11} (0.62)	{16} (− 0.66)	{5, 11, 17} (− 0.77)
	73	{0, 11} (0.62)	{3} (0.84)	{13} (0.94)	{2, 3, 24} (0.38)
	94	{0, 11} (− 0.28)	{11} (− 0.17)	{11} (− 0.17)	{7, 11, 15} (− 0.63)
	143	{8, 24} (0.50)	{8} (0.63)	{17} (0.43)	{8, 17, 26} (− 0.03)
	166	{0, 18} (0.03)	{11} (0.59)	{18} (0.24)	{14, 18} (− 0.32)
	299	{23, 28} (0.57)	{3} (0.78)	{23} (0.84)	{3, 8, 15} (0.28)
	332	{1, 9, 17} (0.20)	{2} (0.65)	{2} (0.65)	{4, 17, 19} (0.39)
	368	{0, 25} (− 0.29)	{28} (0.70)	{29} (− 0.23)	{11, 28, 29} (− 0.65)
*wdbc*	79	{0, 17} (− 0.27)	{8} (0.59)	{16} (− 0.66)	{16, 19} (− 2.20)
	83	{0, 2, 7} (0.56)	{3} (0.79)	{3} (0.79)	{0, 9, 11} (0.19)
	89	{1} (0.31)	{21} (0.22)	{21} (0.22)	{1, 8, 20} (0.05)
	96	{4} (0.66)	{4} (0.66)	{4} (0.66)	{4, 5, 9} (− 0.23)
	103	{8, 10} (0.47)	{12} (0.59)	{12} (0.59)	{12, 14, 27} (0.35)
	104	{0, 11} (− 0.29)	{24} (0.16)	{11} (− 0.17)	{11, 20, 21} (− 0.43)
	153	{4, 8, 14} (0.02)	{8} (0.56)	{17} (0.42)	{9, 17, 28} (0.26)
	176	{1, 18} (− 0.18)	{11} (0.59)	{18} (− 0.10)	{9, 14, 18} (− 1.17)
	271	{8, 9} (0.58)	{8} (0.71)	{11} (0.66)	{4, 8, 10} (0.52)
	309	{0, 8} (0.34)	{3} (0.71)	{23} (0.78)	{2, 4, 8} (0.27)
*wpbc*	2	{0, 1} (0.05)	{0, 4} (0.05)	{0, 4} (0.05)	{0, 1, 19} (− 0.18)
	8	{3, 18} (0.62)	{18} (0.90)	{18} (0.90)	{3, 13, 20} (0.44)
	9	{0, 30} (− 0.38)	{10} (0.54)	{30} (− 0.02)	{9, 30, 31} (− 0.51)
	34	{5, 20} (0.49)	{16} (0.27)	{16} (0.27)	{0, 16, 21} (− 0.37)
	56	{6, 19} (− 0.12)	{10} (0.47)	{19} (0.28)	{26, 28, 29} (0.22)
	58	{0, 5} (− 0.01)	{5} (0.39)	{15} (0.17)	{8, 15, 25} (− 0.65)
	68	{18, 31} (0.21)	{31} (0.59)	{19} (0.62)	{0, 6, 18} (0.71)
	90	{0, 17} (− 0.42)	{15} (− 0.33)	{15} (− 0.33)	{8, 15} (− 0.45)
	119	{1, 31} (− 0.58)	{31} (− 0.02)	{31} (− 0.02)	{6, 21, 31} (− 1.13)
	161	{0, 14} (− 0.75)	{21} (0.30)	{21} (0.30)	{5, 12, 14} (− 0.53)

${{\textbf {q}}}$-id represents query point index; the numbers in the bracket (subspace) are attribute indices

Best results are underlined

**Table 7:**
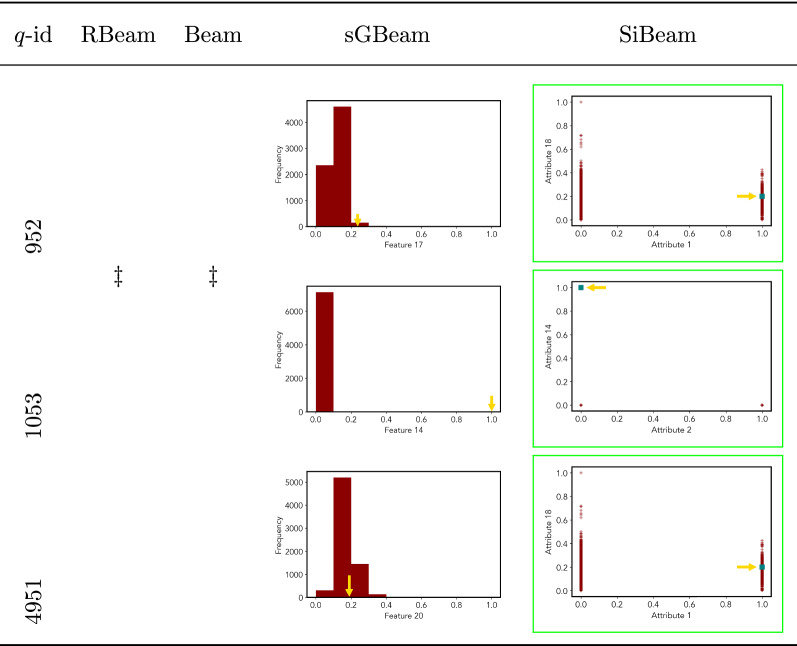
Visualization of discovered subspaces by RBeam, Beam, sGBeam, and SiBeam in the ***annthyroid*** data set

**Table 8:**
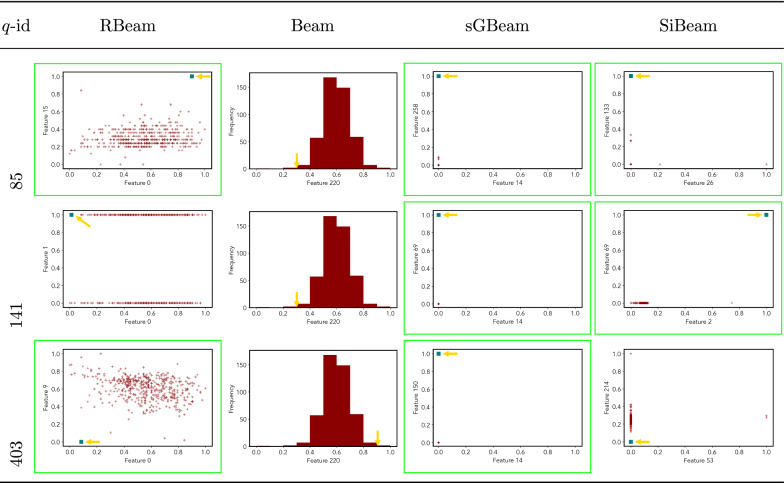
Visualization of discovered subspaces by RBeam, Beam, sGBeam, and SiBeam in the ***arrhythmia*** data set

**Table 9:**
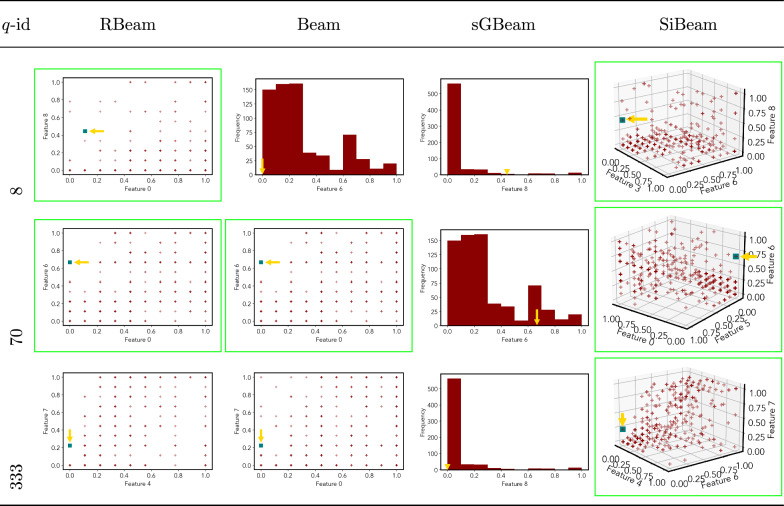
Visualization of discovered subspaces by RBeam, Beam, sGBeam, and SiBeam in the ***breastw*** data set

**Table 10:**
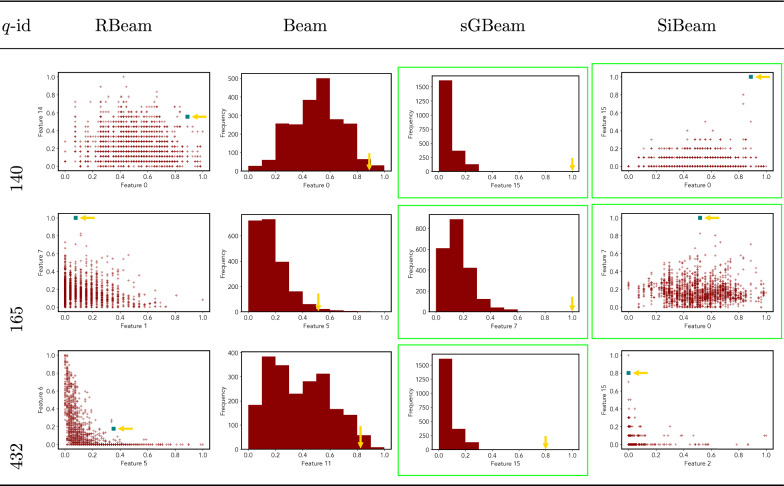
Visualization of discovered subspaces by RBeam, Beam, sGBeam, and SiBeam in the ***cardiotocography*** data set

**Table 11:**
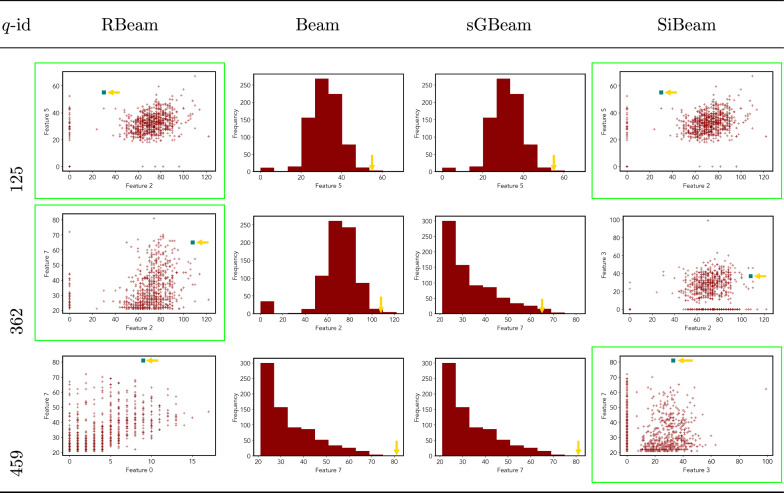
Visualization of discovered subspaces by RBeam, Beam, sGBeam, and SiBeam in the ***diabetes*** data set

**Table 12:**
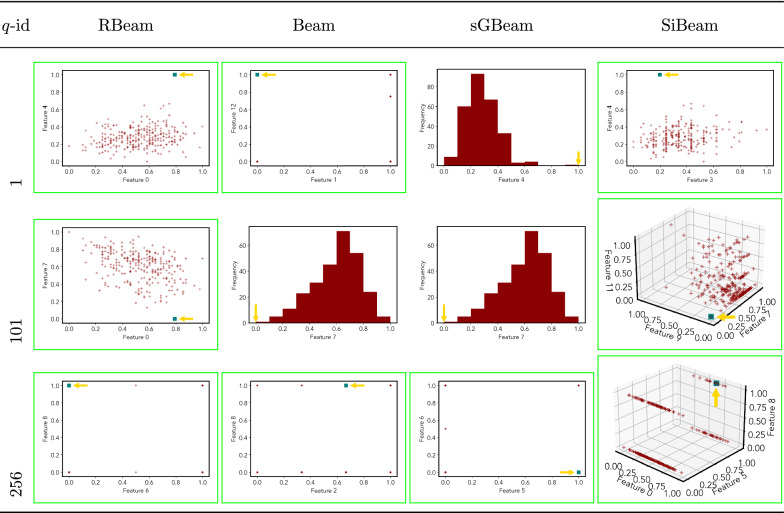
Visualization of discovered subspaces by RBeam, Beam, sGBeam, and SiBeam in the ***heart***_***disease*** data set

**Table 13:**
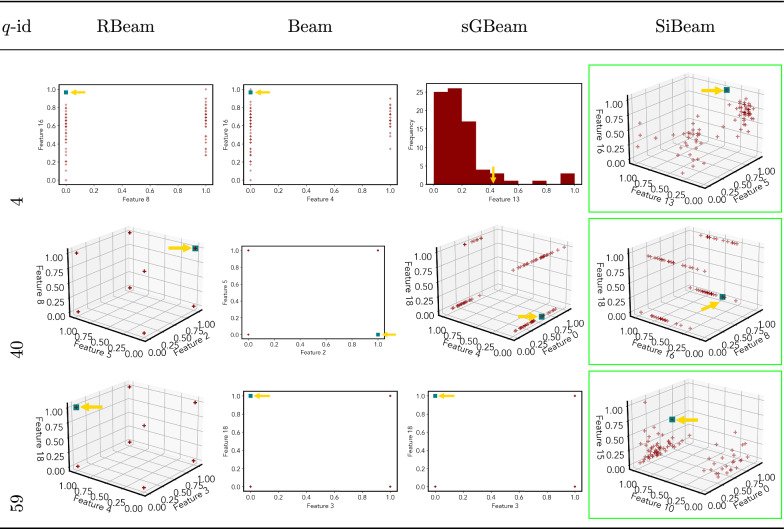
Visualization of discovered subspaces by RBeam, Beam, sGBeam, and SiBeam in the ***hepatitis*** data set

**Table 14:**
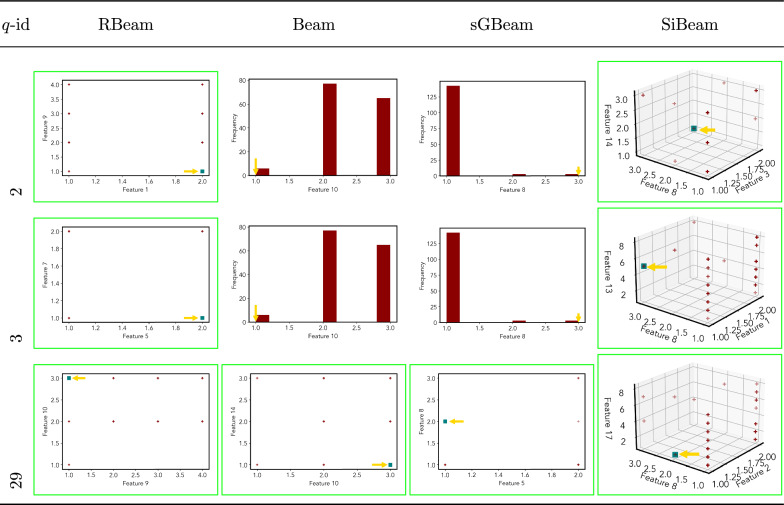
Visualization of discovered subspaces by RBeam, Beam, sGBeam, and SiBeam in the ***lympho*** data set

**Table 15:**
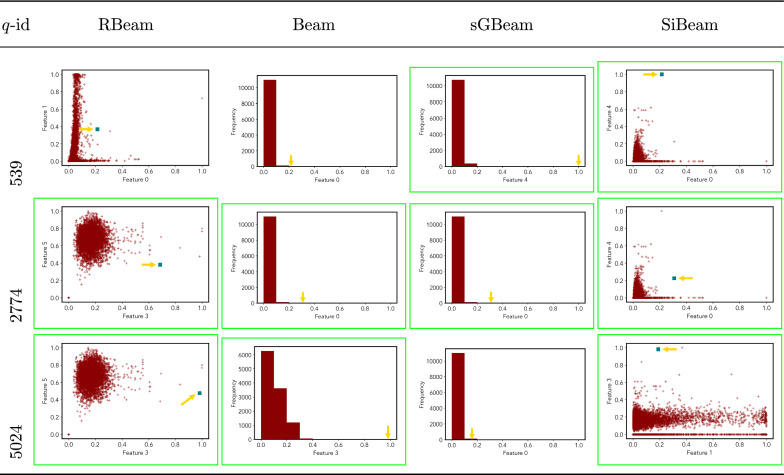
Visualization of discovered subspaces by RBeam, Beam, sGBeam, and SiBeam in the ***mammography*** data set

We first use the iForest anomaly detection method for each data set to detect top *k*=10 anomalies; then, they are used as queries. Each scoring measure identifies outlying aspects for each anomaly (queries). We detect the quality of subspace using Eq. 1.

Tables 3, 4, 5 and 6 shows the subspace found by four scoring measures and quality of discovered subspace on 16 real-world medical datasets. RBeam and Beam cannot finish on *annthyroid* and *musk* in an hour; thus, we presented as ‘$\ddagger$’.

Out of 160 queries, SiBeam detects a better subspace for 116 queries, and sGBeam detects a better subspace for only 23 queries. While RBeam detects better subspaces for 40 out of 140 queries and Beam only for 6 queries. Overall, SiBeam is the best-performing measure, and RBeam is a slow measure; however, it performs better than the *Z*-score-based measure. As mentioned in [20, 21], *Z*-score-based measures are biased towards subspace having high variance. Thus, both *Z*-score-based measures perform worst in this comparison.

Next, we visually present the discovered subspaces by different scoring measures of three queries from each data set. Note that each one-dimensional subspace is plotted using a histogram with 10 equal-width bins.

Tables 7, 8, 9, 10, 11, 12, 13, 14, 15, 16, 17, 18, 19, 20, 21, and 22 provides the visualization of discovered sub-spaces by RBeam, Beam, sGBeam, and SiBeam on *annthyroid*, *arrhythmia*, *breastw*, *cardiotocography*, *diabetes*, *heart disease*, *hepatitis*, *lympho*, *mammography*, *musk*, *pima*, *thyroid*, *vertebral*, *wbc*, *wdbc* and *wpbc* respectively. The query point is highlighted with a dark blue-green (teal) color and a golden color arrow. We highlighted visually better subspace with a green box.

By visually comparing discovered subspaces by each measure, out of 48 queries (3 from each data set), SiBeam and sGBeam detect better subspaces for 39 and 18 queries. In contrast, RBeam and Beam detect better subspaces for 29 and 11 out of 42 queries. Overall, visually we can say that SiBeam performs best or comparative to RBeam, Beam, and sGBeam.

**Table 16:**
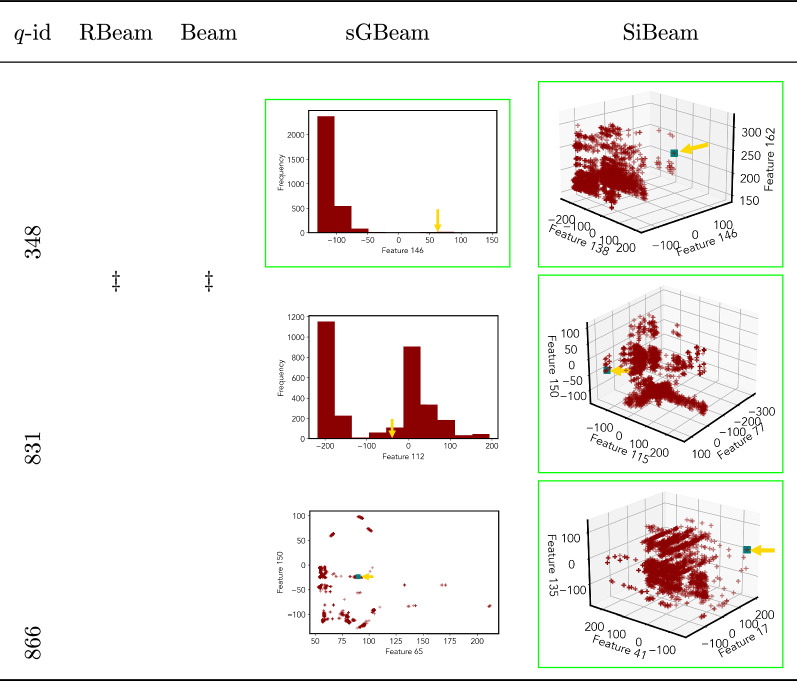
Visualization of discovered subspaces by RBeam, Beam, sGBeam, and SiBeam in the ***musk*** data set

**Table 17:**
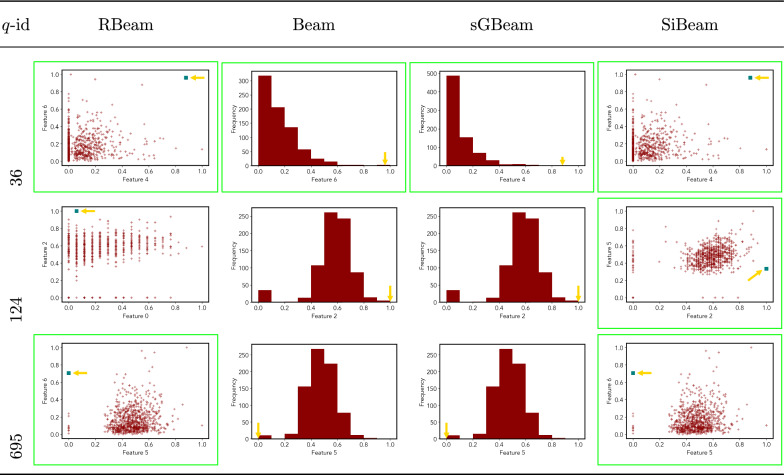
Visualization of discovered subspaces by RBeam, Beam, sGBeam, and SiBeam in the ***pima*** data set

**Table 18:**
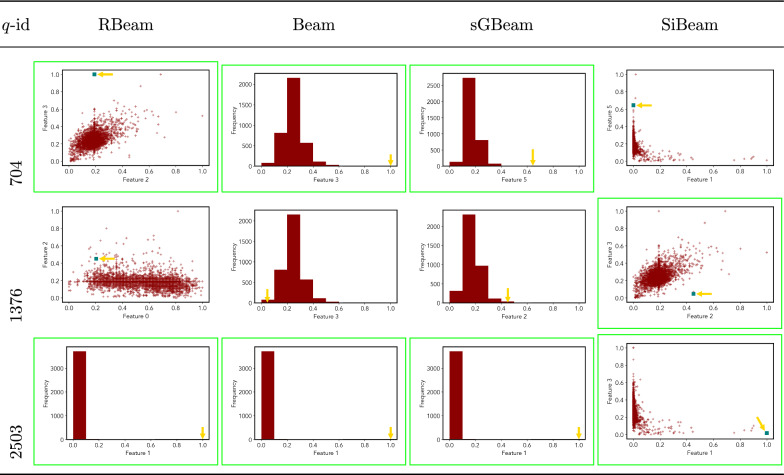
Visualization of discovered subspaces by RBeam, Beam, sGBeam, and SiBeam in the ***thyroid*** data set

## Conclusion

This paper shows an interesting application of OAM in the healthcare domain. We first introduced four anomaly detection and outlying aspect mining algorithms. Then, we presented a framework that not only detects anomalies but also explains why a given query is an anomaly; by providing a set of features where it is most outlying compared to others. Our evaluation on 16 medical datasets shows that iForest is the best-performing measure. Furthermore, our experiment on the task of anomaly explanation (outlying aspect mining) shows that the recently developed isolation-based outlying scoring measure SiNNE outperforms other state-of-the-art outlying aspect mining scoring measures. In the medical domain, it is essential to have a fast algorithm; thus, kernel density or *Z*-score-based scoring measures are not suitable while the data set is huge.

**Table 19:**
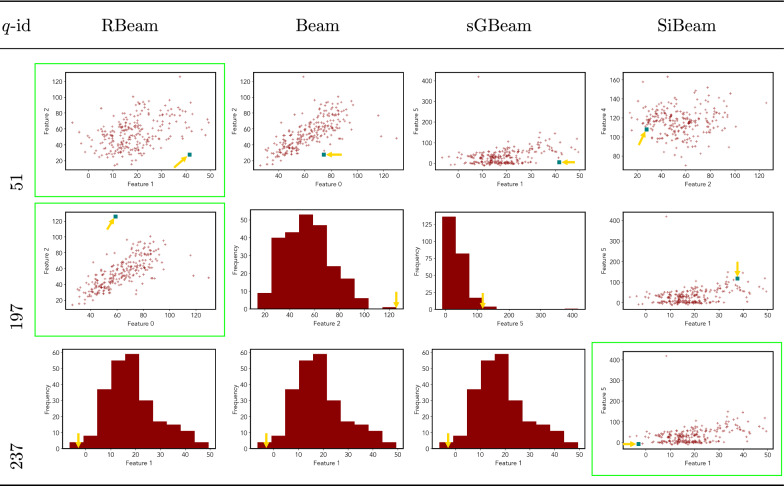
Visualization of discovered subspaces by RBeam, Beam, sGBeam, and SiBeam in the ***vertebral*** data set

**Table 20:**
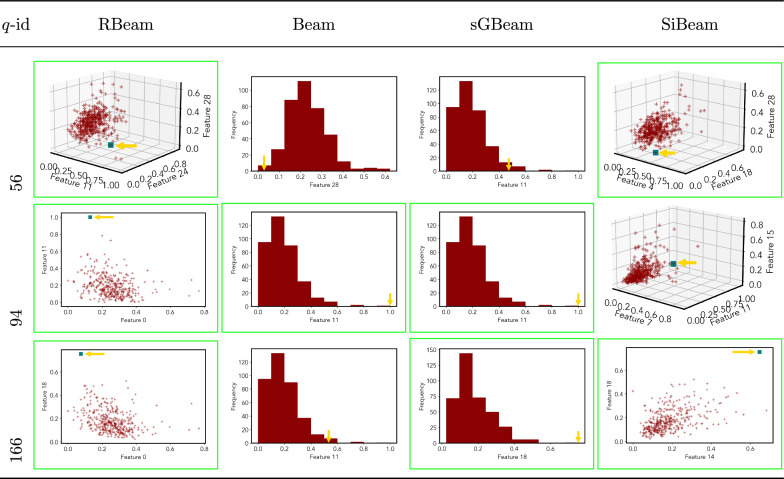
Visualization of discovered subspaces by RBeam, Beam, sGBeam, and SiBeam in the ***wbc*** data set

**Table 21:**
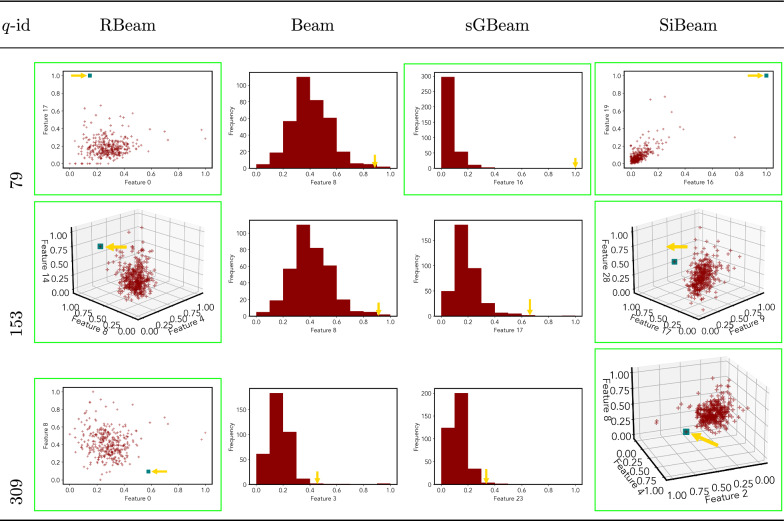
Visualization of discovered subspaces by RBeam, Beam, sGBeam, and SiBeam in the ***wdbc*** data set

**Table 22:**
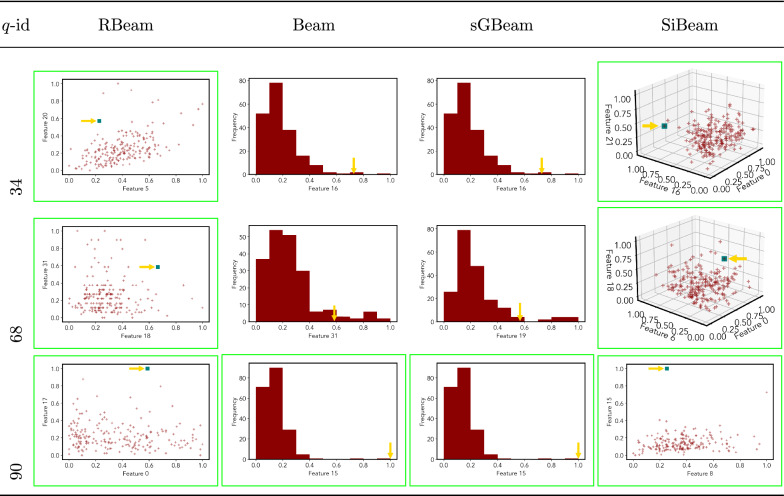
Visualization of discovered subspaces by RBeam, Beam, sGBeam, and SiBeam in the ***wpbc*** data set

## Footnotes

1 Anomaly and outlier are the most commonly used terms in the literature. In this work, hereafter, we will use anomaly term only.

2 The description of the data set is provided in Table 1.

3 Available at https://www.ipd.kit.edu/~muellere/HiCS/

4 Available at https://www.dbs.ifi.lmu.de/research/outlier-evaluation/DAMI/

5 Available at http://odds.cs.stonybrook.edu

6 Note that, hereafter, we denote precision at n by *P*@*n*.
